# Great tits do not compensate over time for a radio‐tag‐induced reduction in escape‐flight performance

**DOI:** 10.1002/ece3.8240

**Published:** 2021-11-12

**Authors:** Barbara M. Tomotani, Florian T. Muijres, Bronwyn Johnston, Henk P. van der Jeugd, Marc Naguib

**Affiliations:** ^1^ Department of Animal Ecology Netherlands Institute of Ecology Wageningen The Netherlands; ^2^ Experimental Zoology Group Wageningen University & Research Wageningen The Netherlands; ^3^ Behavioural Ecology Group Wageningen University & Research Wageningen The Netherlands

**Keywords:** actuator disk model, bird flight maneuverability, experiment, *Parus major*, predation, radio‐tag

## Abstract

The use of biologging and tracking devices is widespread in avian behavioral and ecological studies. Carrying these devices rarely has major behavioral or fitness effects in the wild, yet it may still impact animals in more subtle ways, such as during high power demanding escape maneuvers. Here, we tested whether or not great tits (*Parus major*) carrying a backpack radio‐tag changed their body mass or flight behavior over time to compensate for the detrimental effect of carrying a tag. We tested 18 great tits, randomly assigned to a control (untagged) or one of two different types of a radio‐tag as used in previous studies in the wild (0.9 g or 1.2 g; ~5% or ~6–7% of body mass, respectively), and determined their upward escape‐flight performance 1, 7, 14, and 28 days after tagging. In between experiments, birds were housed in large free‐flight aviaries. For each escape‐flight, we used high‐speed 3D videography to determine flight paths, escape‐flight speed, wingbeat frequency, and actuator disk loading (ratio between the bird weight and aerodynamic thrust production capacity). Tagged birds flew upward with lower escape‐flight speeds, caused by an increased actuator disk loading. During the 28‐day period, all groups slightly increased their body mass and their in‐flight wingbeat frequency. In addition, during this period, all groups of birds increased their escape‐flight speed, but tagged birds did so at a lower rate than untagged birds. This suggests that birds may increase their escape‐flight performance through skill learning; however, tagged birds still remained slower than controls. Our findings suggest that tagging a songbird can have a prolonged effect on the performance of rapid flight maneuvers. Given the absence of tag effects on reproduction and survival in most songbird radio‐tagging studies, tagged birds in the wild might adjust their risk‐taking behavior to avoid performing rapid flight maneuvers.

## Graphical abstract

We tested how escape‐flight performance of great tits was affected by a backpack radio‐tag, and whether or not tagged birds would change their body mass or flight behavior to compensate for carrying a tag. Tagged birds had a larger actuator disk loading and lower escape flight speed than untagged control birds. Moreover, throughout the 28‐day experiment, the escape flight speed increased in both groups but less so in tagged birds, with no tag‐specific body mass or wing beat frequency compensation. Tagging has thus a prolonged effect on the performance of rapid flight maneuvers, and wild birds might adjust via changes in their risk‐taking behavior.

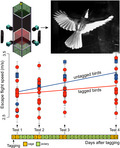

## 1 INTRODUCTION

The use of biologging and tracking devices has become a common and widespread practice when studying animals in the wild. Biologging and tracking devices have opened new doors to understand animal behavior, ecology, and physiology, as they allow collecting data from moving animals in unprecedented ways. Such devices allow tracking individual movements across large time and spatial scales, and often to collect additional behavioral or physiological data, which would be impossible otherwise (Bridge et al., 2013; Wilmers et al., 2015). These include radio and GPS tags (Barron et al., 2010; Snijders, Nieuwe‐Weme, et al., 2017), backpack microphones (Gill et al., 2016), proximity loggers and accelerometers (Chakravarty et al., 2019), depth loggers under water (Meise et al., 2013), or various physiological data, such as activity patterns (Dominoni et al., 2017), heart rate, body temperature (Woakes et al., 1995), sleep (Rattenborg et al., 2016), or metabolites (Gumus et al., 2015). Specifically, tracking devices have revealed intriguing data on dispersal and migratory behavior (e.g., Willemoes et al., 2015), where animals can be followed over thousands of kilometers (Egevang et al., 2010), as well as information at smaller spatial scales, such as social structures within populations using proximity logging or localizing individuals directly (Amrhein et al., 2004; Farine et al., 2015; Snijders et al., 2014) (for further reviews covering the uses of tracking devices, see Bridge et al., 2013; Geen et al., 2019; Hussey et al., 2015; Katzner & Arlettaz, 2020; López‐López, 2016).

Regardless of the specific purpose of a biologging or tracking device, care must be taken so that the data collection has a low impact on the animal. Thus, size, shape, and mass of the device, as well as attachment method and ecological context, need to be considered when applying such devices (Barron et al., 2010; Snijders, Nieuwe‐Weme, et al., 2017). Yet, while many studies, including meta‐analyses, show small, if any, effects of commonly used tracking devices (e.g., Brlík et al., 2020; Costantini & Møller, 2013), even when birds carry them for long periods and across long migratory journeys, it still is inherently difficult to collect data on the actual impact of devices. For example, survival data are one of the most relevant and often most easily collected information, since tracking devices allow determining whether and when an animal stops moving. However, survival data have limitations as there is usually a decision of tagging individuals that are more likely to survive and few studies report the survival rate of control untagged animals (Brlík et al., 2020). For instance, animals disappearing may have either died or moved away from a study population, or tags may have fallen off unnoticed to the researcher. Likewise, survival can be context‐ and individual‐specific: Even if no significant general effect is found, it cannot be excluded that some animals die due to a device impact in a very specific context, such as in dense vegetation or when avoiding a predator. Finally, impacts on an animal can be subtle and, even in the absence of survival effects, they may impact movement, time budget, or the behavior that is being studied.

While the technology in biologging and tracking is advancing at a high pace and effort, specifically with respect to reducing the size of devices (Wilmers et al., 2015), often devices have been larger or heavier than a researcher may wish. Commonly applied rules of thumb of maximum device mass not only vary but are also based on limited studies and then generalized across taxa (Snijders, Nieuwe‐Weme, et al., 2017; Tomotani et al., 2019). While there is a common understanding that the probability of having a negative effect will increase with tag mass, the data are distributed such that a clear convincing rule cannot be scientifically substantiated.

In songbirds, tag mass is usually advised to not exceed a maximum of 5% of the body mass (based on Brander & Cochran, 1969, further tested on bats, Aldridge & Brigham, 1988), yet this “rule” is not grounded on scientific data. The existing data vary substantially, showing no tag effects (Bell et al., 2017; Peterson et al., 2015), effects of tags irrespective of their mass (Bowlin et al., 2010; Tomotani, Bil, et al., 2019), or effects being context rather than mass specific (Atema et al., 2016; Snijders, Nieuwe‐Weme, et al., 2017). Moreover, the time period over which an animal carries a tag might play a role as birds might adapt to the increase in weight. Animals may reduce their body mass over time, for example, when they are molting, and a similar strategy could also be employed by animals when compensating for attachment of devices (Lind, 2001; Lind et al., 2004, 2010). Indeed, birds are capable of varying their body mass substantially, for example, across the day (over 9% difference between morning and evening masses in captive zebra finches, Metcalfe & Ure, 1995) or across seasons (*e*.*g*., prior to migration Lindström, 2003). They also naturally deal with carrying an additional mass such as during reproduction when females carry the developing eggs or predators that need to lift their prey (Kullberg et al., 2002; Lind et al., 2010). Thus, to fully understand the specific effects of a tag on an animal, effects must be measured over a sufficiently long time period, as it is possible that negative impacts over time diminish, specifically in animals that vary naturally in mass across time and contexts. Researcher decisions over which animals to tag may be particularly important in those cases. For example, biomechanical analyses show that the total mass is more relevant than the relative tag mass when body mass is not necessarily correlated with the animal size (*e*.*g*., migratory birds that accumulate large fat reserves). Therefore, choosing the heavier individual in order to minimize relative tag mass may be very misleading because that individual may already be constrained in its flight performance (Tomotani, Bil, et al., 2019).

The specific mechanistic impact of a device on an animal can be subtle and come in different ways: A backpack tag, for instance, can disrupt the force balance of a flying bird by shifting the center of gravity upward and forward or backward, and increase aerodynamic drag by changing the shape of the body (Bowlin et al., 2010; Lind et al., 2010; Pennycuick et al., 2011). Tag attachment thus may not only incur the energy balance due to an increase in energetic cost of locomotion, but it can also affect other aspects of locomotion flight control or escape‐flight speed. Effects may not be immediately visible: Animals may perform without constraints during their daily activities but be impacted when escaping from a predator or when fast flight maneuvers are required during social conflicts, such as territory disputes or inter‐sexual interactions. Thus, the impact of a tag might also depend on predation pressure, social context, and not be detected as statistically significant in studies where predation rates and physical social conflicts are low. Given the challenges of testing the impact of biologging and tracking devices on behavioral performance in the wild, controlled experiments are thus urgently needed.

Here, we thus tested how different types of backpack radio‐tags affect the escape‐flight performance of wild‐caught great tits (*Parus major*), focusing particularly on whether or not these birds adapt their body mass or behavior over time to carrying the tag. Our main research question hereby is whether and how tagged birds adapt their flight behavior or morphology over time to reduce the potential detrimental effect of tagging on escape‐flight performance. We used escape‐flight performance as a proxy for rapid flight maneuverability, which is relevant in many natural flight behaviors such as in‐flight hunting, predator evasion, during social conflicts such as territory disputes, or inter‐sexual interactions such as flight display. We predicted that tagged birds would have a slower escape‐flight speed than controls and that birds with heavier tags would have a lower escape‐flight performance than birds with lighter tags. Finally, we expected that tagged birds would reduce their body mass over time to compensate for the added tag mass, thus increasing their escape‐flight speed more rapidly than untagged control birds.

We equipped wild‐caught birds with either no radio‐tag, a 0.9 g radio‐tag, or a 1.2 g radio‐tag, for convenience called the lighter and heavier tag, respectively. Both tag types have been used in previous studies in the wild (Bircher et al., 2020, 2021; Snijders et al., 2014, 2017). In those studies, no direct effects of tag mass on breeding and parental care were found (Snijders, Nieuwe‐Weme, et al., 2017). After tag deployment and throughout the experimental period of one month, we housed all birds in free‐flight aviaries, without removing the radio‐tags. At set intervals within this month, we determined the escape‐flight performance of all birds via flight experiments. In each trial, a bird would fly upward in a vertical flight tunnel while being filmed using multiple synchronized high‐speed cameras. Based on the stereoscopic video recordings, we reconstructed the three‐dimensional flight trajectory and determined flight speed and wingbeat frequency during the escape flight. We modeled the functional effect of tag weight on escape‐flight performance based on the tag‐induced increase in actuator disk loading (Muijres et al., 2011; Rayner, 1979), defined as the body (and tag) weight per unit area of a circular actuator disk with a diameter equal to the wingspan. This actuator disk load quantifies the ratio between the weight of flying bird and its ability to produce an upward‐directed aerodynamic thrust force, which scales with actuator disk area (Muijres et al., 2011; Rayner, 1979). Thus, a heavier bird, a tagged bird, or a bird with smaller wings will have a relatively larger disk loading and consequently would need to invest more in order to rapidly fly upward compared to a lighter, untagged, or larger‐winged conspecific. Using this model, we tested how escape‐flight performance was affected by the weight of the device, and how birds adapted to the tag during a month of deployment.

## 2 METHODS

### 2.1 Animals and housing

We captured wild great tits using mist nets and playback recordings between November 1 and 2, 2017, in Wageningen, the Netherlands (51.9692°N, 5.6654°E). Birds were immediately taken to the Netherlands Institute of Ecology (NIOO‐KNAW), which is close to the capture sites, and housed in same‐sex groups in two large outdoor aviaries (4.2 × 1.9 × 2.1 m). For each of the flight tests, we transferred the birds to indoor individual cages (90 × 50 × 40 cm) one day before the test and then transferred them back to the aviaries one day after the test (see below). While in captivity, in both aviaries and cages, birds had access to food (insects and seeds) and water ad libitum. While in aviaries, they had also access to nest boxes for roosting. Birds were tagged five days after their capture in the wild and, after approximately 45 days in captivity, tags were removed and birds were released by opening the outdoor aviary doors.

### 2.2 Experimental design

For the study, we used 18 great tits (nine females, nine males, all first calendar year birds) that were randomly assigned to one of three treatments: (a) a control treatment, where birds were handled and tested like the radio‐tagged individuals, but the tags were not deployed, (b) a treatment where birds were deployed with the relatively lighter tag (0.9 g), and (c) a treatment where birds were deployed with the relatively heavier tag (1.2 g). The masses of the relatively lighter and heavier tags (*m*_tag_) represented approximately 6% and 7.5% of the birds’ body masses (*m*_bird_), respectively (females with lighter tag: *m*_tag_/*m*_bird_ = 0.063 ± 0.0002 (mean ± standard error), *n* = 3 birds; males with lighter tag: *m*_tag_/*m*_bird_ = 0.058 ± 0.0001, *n* = 3 birds; females with heavier tag: *m*_tag_/*m*_bird_ = 0.078±0.0005, *n* = 3 birds; males with heavier tag: *m*_tag_/*m*_bird_ = 0.070±0.0009, *n* = 3 birds). Both types of tags had an antenna and were mounted using leg loop harnesses. Tags were the exact same models as those previously used in the field (Bircher et al., 2020; Snijders et al., 2014), and for neither of them, negative fitness effects were observed (Snijders, Nieuwe‐Weme, et al., 2017). As a result, we had three females in the control treatment, three females in the lighter tag and three females in heavier‐tag treatments and three males in the control treatment, three males in lighter‐tag treatment and three males in the heavier tag treatments.

**FIGURE 1:**
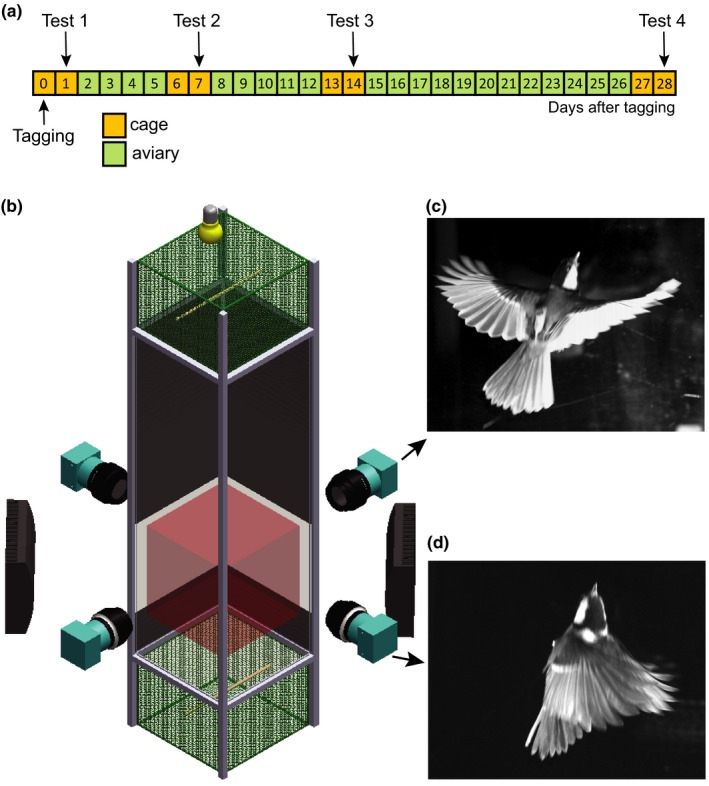
Experimental design and flight setup. (a) Experimental design showing the moments that birds were housed in cages or aviaries and in which days the experimental flight tests took place. (b) Schematic representation of the experimental setup: a vertical flight tunnel with four high‐speed cameras, infrared illumination (black light panels), and a release box with perch at the bottom and a collection box with perch at the top. The collection box is illuminated with visible light. The focal area where flight was recorded is shown in red. (c, d) Cropped example images captured by different cameras of the same bird, showing an upward flying tagged bird during mid‐downstroke (c) and at the start of the downstroke (d)

To assess whether a potential radio‐tag effect would disappear over time due to habituation, birds from all treatments were tested on four different days, being *T* = 1, 7, 14, and 28 days after tag deployment (Figure 1a). In the afternoon of the day before each test, birds were moved from the outdoor aviaries to the indoor cages. Tests then took place in the following morning (between 8:00 h and 12:00 h); one day after the testing day, birds were moved back to the aviaries. The only exception was the test on day 1 as, after tagging, birds were kept in the small cage and tested in the following day, without spending any time in the aviary (Figure 1a). Keeping the birds in small cages prior to the flight test was important to standardize capture and handling time of each individual prior to the test (which was on average <5 min per bird). By keeping birds in aviaries in between testing days, we allowed the birds to fly, interact with the other individuals, and adapt to the radio‐tags. No bird experienced perceptive feather damage from capture in neither aviaries nor cages. Moreover, by using the same procedure for all individuals, we controlled for eventual handling effects so they were similar across all experimental groups.

### 2.3 Flight performance trials and parameters

To collect data on flight performance, we used an experimental setup consisting of a transparent vertical flight tunnel measuring 50 × 50 × 210 cm (width × depth × height) and equipped with two interchangeable 30 cm high boxes: a release box on the bottom of the flight tunnel, and a collection box on the top, both equipped with a perch (Figure 1b; Tomotani, Bil, et al., 2019; Tomotani & Muijres, 2019; Tomotani et al., 2018).

Prior to each trial, a single bird was weighed and then placed inside the release box. The release box with the bird inside was then transported to the flight setup and placed into the bottom of the setup. Before the test, all lights were turned off so that the whole room was kept in complete darkness while the release box was carefully opened. We then elicited the escape flight by turning on a single light bulb illuminating the perch in the collection box at the top of the flight tunnel. This caused birds to fly upward toward this perch and land on it. We then closed the lid of the collection box and swapped the release and collection boxes, allowing us to repeat the procedure without having to handle the bird. On each test day, we repeated the upward flight experiment for each individual bird successively five times. The whole procedure took around 20 min per bird per test day.

### 2.4 Flight recording and data processing

We filmed birds flying upward across a section of approximately 40 × 40 × 40 cm within the bottom half of the setup using a stereoscopic videography system (Figure 1b, Movies S1–S2). This system consisting of four synchronized high‐speed cameras (Mikrotron EoSens MC1362) recording at 400 frames per second. Each camera had a spatial resolution of 1020 × 1020 pixels and an exposure time of 1 millisecond. We used infrared lights (Bosch Aegis SuperLed 850 nm) to illuminate the recording area, whereas a visible light source (40 W incandescent light bulb) illuminated the collection box (Figure 1b). The experimental room in which the setup was placed was otherwise kept completely dark during all experiments.

At the start of each experimental day, we calibrated the stereoscopic camera system by placing a calibration grid in the setup and filming it. The calibration grid consisted of a randomly distributed array of lead beats on strings, covering the complete filming volume. We manually tracked the position of all beats in each camera view, and based on these, we calibrated the camera system using a DLT calibration routine (Hatze, 1988).

We analyzed the upward escape maneuvers by manually tracking the birds using the DTLdv digitizing tool in MATLAB (MathWorks Inc.) (Hedrick, 2008). In each recording, we manually tracked the beak of the bird in all camera views, and we determined the start of the downstroke of each wingbeat. Based on the downstroke data, we determined the trajectory‐average wingbeat frequency *f* of the upward‐flying bird. The location of the beak was used to estimate the escape‐flight speed in each maneuver. We used beak position for this because the beak was easy to identify in each camera view, and because it oscillated relatively little as a result of the flapping wingbeat movements (Movies S1–S2). Using the camera calibration, we converted the stereoscopic camera tracks of the beak into a three‐dimensional flight path **X**(*t*), which was then Kalman filtered to remove tracking noise and estimate flight velocity throughout the flight trajectory **U**(*t*). From the filtered track data, we estimated the trajectory‐average flight speed *U* (Figure 1c,d). For one video recording per individual, we also tracked the wing tip movement throughout at least one wingbeat. Based on these data, we determined the maximum wingspan *b*_max_ during flight of that bird (Figure 1).

### 2.5 Functional actuator disk model for upward flight

To maintain its flight speed, an upward escaping bird needs to produce an upward‐directed aerodynamic thrust force equal to its weight. The bird produces this thrust force by beating its wings. Most passerine birds produce the majority of these forces during the downstroke, as the upstroke is aerodynamically mostly inactive (Crandell & Tobalske, 2015; Muijres et al., 2012; Tomotani & Muijres, 2019). During the downstroke, the bird beats its wings in an inclined arc, thereby accelerating air downward within a disk spanned by the wingtip paths, and consequently produce the upward‐directed thrust. Therefore, aerodynamic thrust force production for slow‐flying birds is often modeled using actuator disk theory, which has been developed to model thrust force production of propeller rotor systems (Muijres et al., 2011, 2012; Rayner, 1979).

Here, we propose to use this model to estimate the thrust production capacity of our upward‐flying birds. According to the actuator disk theory, thrust production capacity of a bird scaled linearly with the surface area of the actuator disk. We modeled the actuator disk of our upward‐flying birds as a circular disk with diameter equal to the maximum wingspan during the wingbeat downstroke (Figure 1), and thus actuator disk area *A* is defined as

(1) A=14πbmax2,

where *b*_max_ is the maximum wingspan throughout a wingbeat, determined from the videography data. Based on this area, we then defined the actuator disk loading as

(2) $$\frac{W}{A}=\frac{4W}{\pi b_{\max}^{2}},$$

where *W* is the weight of the bird in Newtons and determined as the product of body mass *m* and gravitational acceleration (*g* = 9.81 m/s^2^). This disk loading scales with the ratio between the downward‐directed weight vector and the upward‐directed vector of the thrust force that the bird can produce. Thus, a lower disk loading would allow a bird to fly faster upward; adding a tag to a bird, would increase the disk loading, and consequently reduce escape‐flight speed.

To test the effect of tag attachment on disk loading and escape‐flight speed, we determined two types of disk loading: (a) disk loading based on the bird mass alone *W*_bird_/*A*, and (b) disk loading based on the total mass of the bird and tag combined *W*_bird+tag_/*A*. The first “fictive” disk loading (*W*_bird_/*A*) shows how variations in body mass between individuals and with time affect disk loading. The second “real” disk loading (*W*_bird+tag_/*A*) quantifies the combined effect of adding the tag and changes in body mass on disk loading.

### 2.6 Data analysis

All statistical analyses were carried out in R version 3.6.1 (R Core Team, 2021). All means and parameter estimates are given as mean ± standard error (*n* = sample size in number of birds or flights, depending on the analysis).

First, we tested how the actuator disk loading was affected by our treatments. The actuator disk loading is dependent on both wing size and body mass. Thus, the disk loading increases by adding a radio‐tag. By decreasing its body mass, an individual bird can also reduce its actuator disk loading over time. Both body mass and wing size are known to differ between males and females. Thus, we also compared the male and female differences in their actuator disk loading across treatments. We used mixed‐effect models with treatment (control, lighter tag, heavier tag), day of the test (*T* = 1, 7, 14, and 28 days after tag deployment), and the sex (male, female) as fixed effects. We also fitted a three‐way interaction between treatment, sex, test date as well as the respective two‐way interactions. Finally, because individuals were tested more than once in a test day, we also included the individual bird as a random effect.

Then, we tested whether flight speed was affected by the treatments and whether it would change over time with the tagged birds eventually attaining the same speed as untagged birds. We used mixed‐effect models with either natural or manipulated disk loading (*W*_bird_/*A* or *W*_bird+tag_/*A*, respectively) which account for both effects of wing length and changes in body mass, treatment (control, lighter tag, heavier tag), and day of the test (*T* = 1, 7, 14, and 28 days after deployment) as fixed effects and the three‐way interaction between disk loading, treatment, and test date as well as the respective two‐way interactions and the individual bird as a random effect. Because of the collinearity between sex and disk loading, we did not include sex in this analysis.

Finally, we investigated whether and how birds would compensate for the added mass over time using changes in their wingbeat frequency (skill learning). Once more, we used mixed‐effect models with either natural or manipulated disk loading, treatment (control, lighter tag, heavier tag), and day of the test (*T* = 1, 7, 14, and 28 days after deployment) as fixed effects and the three‐way interaction between disk loading, treatment, and test date as well as the respective two‐way interactions and the individual bird as a random effect.

We also carried out post hoc tests to study specific aspects related to the adjustment of birds to tags: (a) We tested whether natural disk loading (*W*_bird_) changed over time when birds were carrying a device, independently on the device mass, by comparing tagged birds (lumping lighter + heavier treatments) with untagged birds (control). For this, we used a model containing sex, treatment (tagged or untagged), test date, the interaction between test date and treatment and individual as random effect; (b) we also tested for the effects of the tag mass alone on flight speed, by testing for differences in flight speed between pairs of treatments (control–lighter, control–heavier, lighter–heavier) without accounting for test date or disk loading. We used a model with only the treatment described above and individual as random effect; (c) finally, we assessed the effects of carrying a device independently on its mass on flight speed by testing for differences in flight speed between tagged birds (lighter + heavier) and untagged birds (control). We first tested for the effects of tag masses alone without accounting for test date and disk loading in a model containing only treatment (tagged or untagged) and individual as random effect. Then, we tested whether birds carrying a tag differed in performance over time from untagged birds by using a model containing treatment (tagged or untagged), test date, manipulated disk loading (*W*_bird+tag_), the interaction between test date and treatment and individual as random effect.

## 3 RESULTS

### 3.1 Differences in actuator disk loading between individuals and sexes (Appendix Tables A1 and A2)

**FIGURE 2:**
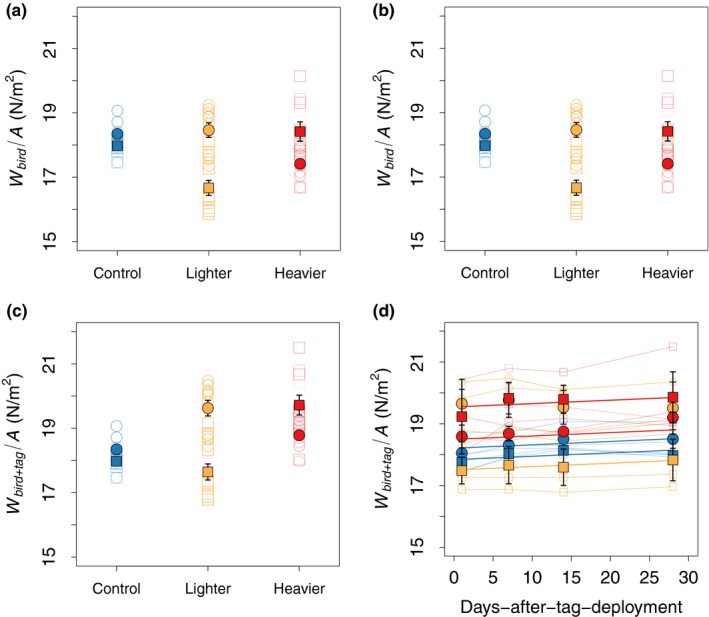
Actuator disk loading in relation to sex, treatment, and time. (a, b) Actuator disk loading based on bird mass only, defined as the ratio between bird weight and actuator disk area (*W*_bird_/*A*). (c, d) Disk loading based on the total mass of bird and tag combined (*W*_bird+tag_/*A*). (a, c) Disk loading for each treatment. (b, d) Changes in disk loading with time (days after tag deployment). In each panel, circles show data for females and squares are for males. Blue data show results of the control group, orange data are of the lighter‐tag group, and red shows data of the heavier‐tag group. Closed symbols with error bars show the mean and standard error for all birds within its group, and open symbols show results of the separate flight sequences. (b, d) Trendlines are predictions of the statistical model (see results)

Males had, on average, a lower disk loading than females (males: *W*_bird_/*A* = 17.70 ± 0.35 N/m^2^, *n* = 9 birds; females: *W*_bird_/*A* = 18.03 ± 0.27 N/m^2^, *n* = 9 birds; Appendix Tables A1 and A2). Males had both a higher body mass *m*_bird_ and a larger maximum wing span *b*_max_ than females. The effect of the wider wing span in males on disk loading was larger than the mass effect on disk loading (Equation 2), which resulted on average in a lower actuator disk loading for the males (Figure 2).

This sex difference in disk loading, however, was not consistent between treatments (interaction between treatment and sex *F*_2,11.99_ = 6.29, *p* = .01, Figure 2a). In both the control treatment and lighter‐tag treatment, the disk loading based on bird mass alone *W*_bird_/*A* was higher for the females (control females: *W*_bird_/*A* = 18.35 ± 0.28 N/m^2^, *n* = 3 birds; control males: *W*_bird_/*A* = 18.00 ± 0.05 N/m^2^, *n* = 3 birds; lighter‐tag females: *W*_bird_/*A* = 18.49±0.48 N/m^2^, *n* = 3 birds; lighter‐tag males: *W*_bird_/*A* = 16.66 ± 0.52 N/m^2^, *n* = 3 birds). In contrast, the heavier‐tag treatment had a reverse pattern, as here females had a lower disk loading than males (heavier‐tag females: *W*_bird_/*A* = 17.24 ± 0.28 N/m^2^, *n* = 3 birds; heavier‐tag males: *W*_bird_/*A* = 18.45 ± 0.63 N/m^2^, *n* = 3 birds). Note that this difference in disk loading based on bird mass alone *W*_bird_/*A* was not caused by the radio‐tags, but was due to the variation in size and body mass among individuals before they were tagged (Figure 2c).

These sex differences were also apparent in the disk loading after tagging (combined mass of bird and tag) *W*_bird+tag_/*A* (Figure 2b): while in the majority of cases, the additional device weight caused the birds in lighter‐tag and heavier‐tag treatments to have a larger disk loading than controls, males on the lighter‐tag treatment ended up with a similar disk loading to controls (Figure 2b; lighter‐tag females: *W*_bird+tag_/*A* = 19.65 ± 0.52 N/m^2^, *n* = 3 birds; lighter‐tag males: *W*_bird+tag_/*A* = 17.63 ± 0.56 N/m^2^, *n* = 3 birds; heavier‐tag females: *W*_bird+tag_/*A* = 18.58 ± 0.31 N/m^2^, *n* = 3 birds; heavier‐tag males: *W*_bird+tag_/*A* = 19.74 ± 0.64 N/m^2^, *n* = 3 birds).

Finally, birds in all treatment groups and of both sexes changed their disk loading similarly over time, with a small but significant increase in disk loading from days 1 to 28 (slope d(*W*_bird_/*A*)/d*T* = 0.01 ± 0.003 N/m^2^ per day, *F*_1,48.19_ = 12.47, *p* < .01, Figure 2b,d). Moreover, the post hoc test comparing birds with and without tags revealed only a date effect (Figure 5b), also indicating a similar increase over time in disk loading across all treatments (slope d(*W*_bird_/*A*)/d*T* = 0.01 ± 0.003 N/m^2^ per day, *F*_1,48.14_ = 12.40, *p* < .01). Because wing span did not change throughout the experiments, these changes in disk loading serve as a proxy for changes in body mass. Thus, birds in all groups had an apparent increase in body mass at a similar rate over time (Figures 2b, d and 5b).

### 3.2 Tagging‐effect and time‐effect on escape‐flight speed (Appendix Tables A2–A4)

#### 3.2.1 Flight speed depends on actuator disk loading

**FIGURE 3:**
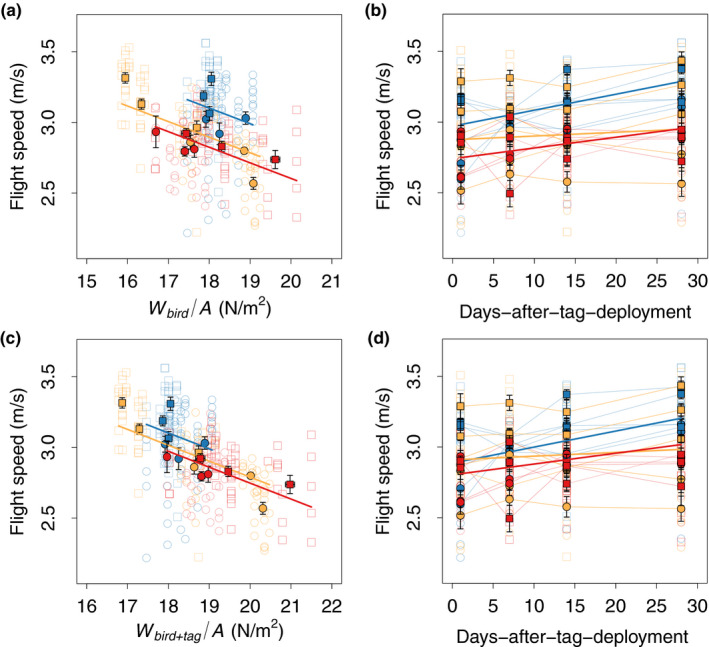
Flight speed in relation to treatment, actuator disk loading, and time. (a, b) Flight speed versus disk loading (a) and time (days after tag deployment) (b), whereby disk loading is based on bird mass only (*W*_bird_/*A*). (c, d) Flight speed versus disk loading (c) and time (days after tag deployment) (d), whereby disk loading is based on the total mass of bird and tag combined (*W*_bird+tag_/*A*). In each panel, circles show data for females and squares are for males. Blue data show results of the control group, orange data are of the lighter‐tag group, and red shows data of the heavier‐tag group. Closed symbols with error bars show the mean and standard error for all birds within its group, and open symbols show results of the separate flight sequences. Trendlines are predictions of the statistical model (see results)

In our vertical flight tunnel, male and female great tits flew upward on average at similar speed (overall means across all treatments: females: *U* = 2.86 ± 0.05 m/s, *n* = 9 birds; males: *U* = 3.05 ± 0.06 m/s, *n* = 9 birds), and the differences in flight speed among individuals and sexes are best explained by differences in actuator disk loading (Appendix Tables A2, A3, A4; Figure 3a,c). Regardless of sex, birds with a larger disk loading (i.e., higher mass‐to‐wingspan‐squared ratio, Equation 2) flew slower than birds with smaller disk loading (Figure 3a: slope d*U*/d(*W*_bird_/*A*) = −0.11 ± 0.03 m^2^ s/kg, *F*_1,27.97_ = 14.74, *p* < .01; Figure 3c: slope d*U*/d(*W*_bird+tag_/*A*) = −0.11 ± 0.03 m^2^ s/kg, *F*_1,27.97_ = 18.03, *p* < .01).

**FIGURE 4:**
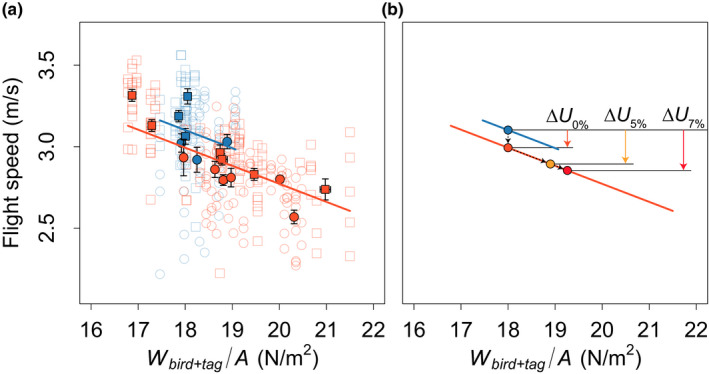
Escape‐flight speed in relation to actuator disk loading and treatment. (a) Flight speed versus disk loading for control birds (blue) and tagged birds (orange). For tagged birds, disk loading is based on bird and tag mass combined (*W*_bird+tag_/*A*). Circles show data for females and squares are for males. Closed symbols with error bars show the mean and standard error for all birds within its group, and open symbols show results of the separate flight sequences. Trendlines are predictions of the post hoc statistical model (see results). (b) Statistical model predictions of the reduction in flight speed as a result of tagging an average bird with *W*/*A* = 18 N/m^2^ and a tag‐free escape‐flight speed of 3.10 m/s (blue circle). We show three scenarios: 1. The bird is tagged with a fictive zero mass tag (0% of body mass), causing an escape‐flight speed reduction of Δ*U*_0%_ = 0.11 m/s (orange circle); 2. tagging the bird with a lighter tag of 5% body mass causes an escape‐flight speed reduction of Δ*U*_5%_ = 0.21 m/s (yellow circle); and 3. tagging the bird with a heavier tag of 7% body mass causes an escape‐flight speed reduction of Δ*U*_7%_ = 0.25 m/s (red circle). For the 5% and 7% tag mass cases, the speed reduction consists of two components: a mass‐independent speed reduction (equal to Δ*U*_0%_) as highlighted by the vertical dashed arrow), and a mass‐dependent component as highlighted by the dashed arrow along the sloped orange line

The post hoc test comparing pairs of treatments without accounting for test date or disk loading showed that the control birds flew up on average significantly faster than birds in the heavier‐tag group, but the escape‐flight speed in the lighter‐tag group did not differ significantly from that of both other groups (Figure 3c, U of control vs. heavier tag: *F*_1,9.74_ = 15.78, *p* < .01, *U* of control vs. lighter tag: *F*_1,10.00_ = 1.51, *p* = .25, *U* of heavier tag vs. lighter tag: *F*_1,9.96_ = 0.87, *p* = .37). Moreover, when birds from the two tagged groups were compared together relative to controls (tagged vs. untagged), untagged birds were on average also significantly faster in flying upward than tagged birds (Figure 4a, of untagged vs. tagged: *F*_1,296.35_ = 5.04, *p* = .04), but in tagged and control birds the relationship between escape speed and disk loading had the same slope (Figure 4a: slope d*U*/d(*W*_bird+tag_/*A*) = −0.11 ± 0.03 m s kg^−1^, *F*_1,25.23_ = 18.95, *p* < .01).

Thus, the differences in flight speed between control and tagged birds were caused by two effects of tagging (Figure 4a,b): the escape‐flight speed in a tagged bird was reduced due to a tag mass‐independent effect, and a mass‐dependent effect caused by the increase in actuator disk loading. The differences in effect of lighter and heavier tags are thus expressed by variation in disk loading *W*_bird+tag_/*A* and not necessarily by the relative mass of the lighter or heavier tag. As a result, birds in the untagged group flew upward on average 6% faster than birds in the tagged group (untagged: *U* = 3.08 ± 0.20 m/s, *n* = 6; tagged: *U* = 2.89 ± 0.20 m/s, *n* = 12).

#### 3.2.2 Flight speed increased with time for all treatment groups

In all groups, the average flight speed increased over time throughout the 28 days of testing (Figure 3b,d). Yet, there was a significant interaction between treatment and test date (*F*_2,297.61_ = 5.49, *p* < .01), indicating that birds in the different treatments changed their flight speed differently over time. Control birds and birds with heavier tags increased their speed over time more rapidly than birds with lighter tags (Figure 3b,d; control: slope d*U*/d*T* = 0.010 ± 0.002 m/s per day; lighter tag: slope d*U*/d*T* = 0.003 ± 0.003 m/s per day; heavier tag: slope d*U*/d*T* = 0.007 ± 0.003 m/s per day). Thus, during the 28 days of testing, control birds increased flight speed the most (8.70%), followed by birds with heavier tags (6.03%). The lighter‐tag birds had the lowest speed increase during the experiment (1.29%).

**FIGURE 5:**
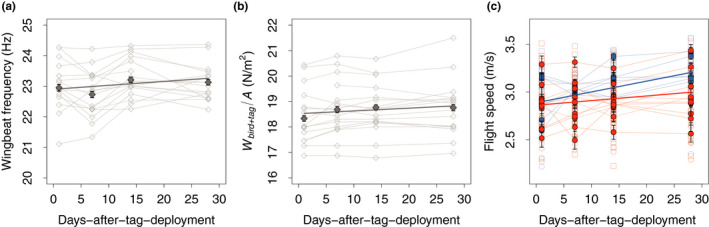
Adjustments over time (days after tag deployment) in (a) wingbeat frequency, (b) bird and tag mass via changes in the corresponding disk loading, and (c) escape‐flight speed for tagged and untagged birds. (a, b) The dark gray line represents the model estimate for the change in time of wingbeat frequency or disk loading as a proxy for weight. Solid symbols with error bars show the mean and standard error of wingbeat frequency for all birds during that day. Open symbols show the mean wingbeat frequency for each individual. (c) Flight speed versus time for control birds (blue) and tagged birds (orange). For tagged birds, weight is based on bird and tag mass combined (*W*_bird+tag_). Circles show data for females and squares are for males. Closed symbols with error bars show the mean and standard error for all birds within its group, and open symbols show results of the separate flight sequences. Trendlines are predictions of the post hoc statistical model (see results)

Moreover, when we compared the flight speed increase over time between tagged and untagged birds, we found that untagged birds increased their flight speed about twice as fast as tagged birds (Figure 5c; untagged birds: slope d*U*/d*T* = 0.010 ± 0.002 m/s per day, tagged birds: slope d*U*/d*T* = 0.005 ± 0.001 m/s per day; *F*_1,296.35_ = 8.09, *p* < .01). Thus, while at the first day the predicted difference in flight speed between untagged and tagged birds was only 1%, it increased to 7% at day 28 (Figure 5c). Thus, both control and tagged birds showed the ability to increase their escape‐flight speed when adapting to the flight cage, but tagged birds increased their escape speed less rapidly than control birds.

### 3.3 Variations in flight behavior: wingbeat frequency increased with time, independent of tagging (Appendix Table A5)

To power their upward flight maneuvers, male and female birds flapped their wings at similar wingbeat frequencies (females: *f* = 23.15 ± 0.24 s^−1^, *n* = 9 birds; males: *f* = 22.98 ± 0.16 s^−1^, *n* = 9 birds; Appendix Tables A5). Wingbeat frequency increased over time equally among all groups (slope d*f*/d*T* = 0.010 ± 0.004 s^−1^ per day; *F*_1,297.31_ = 10.61, *p* < .01), or 1.2% increase in frequency in the 28‐day period of testing (Figure 5). Despite an increase in both disk loading and wingbeat frequency over time, there was no effect of disk loading or treatment on wingbeat frequency. This is probably due to the relatively large variation in disk loading among individuals, compared to the variation in disk loading over time.

## 4 DISCUSSION

Here, we show that the speed of upward escape flights was significantly affected by the birds’ actuator disk loading, which is related to the ratio of body mass and wing span. By equipping birds with the same type of radio‐tags previously used in field studies, we caused an increase in disk loading, which consequently reduced the birds' escape‐flight speed. In addition, next to this tag mass‐dependent effect, we also found a tag effect on escape‐flight speed that was independent of tag mass. Both effects combined significantly impacted the escape‐flight speed of tagged birds, resulting in an average 6% reduction in escape‐flight speed of tagged birds compared to untagged control birds. Both tagged and untagged control birds increased their escape‐flight speed over time, showing that both learned over time to fly upward more quickly in the setup. Yet, tagged birds did not increase their flight speed as rapidly as untagged birds. In fact, directly after tagging, the tagged birds flew upward similarly fast as the untagged birds, but over time a difference in flight speed started to emerge between the groups as the untagged group increased their flight speed more rapidly than the tagged birds.

### 4.1 Escape‐flight speed in tagged birds: morphological and behavioral adjustments over a 28‐day period

Given that escape‐flight speed depended on disk loading, birds could increase their flight speed by reducing their own body mass and thereby decreasing disk loading. In fact, several previous studies have shown that birds can use body mass modulations to adapt to carrying additional mass in terms of food, fat, or eggs (Kullberg et al., 2002; Lind et al., 2010). Alternatively, birds could compensate for the additional weight by changing their flight behavior in order to improve their escape‐flight performance over time. Yet, in our experiment we did not observe such compensatory decrease in body mass. Instead, all treatment groups increased their body mass with a small daily average increase of 0.06% of their initial body mass. This resulted in a 2% overall gain in mass on average during the 28‐day experiment. Although small, this gain in mass directly increased the disk loading, but there were no observable negative consequences to the birds' escape‐flight speed over time.

The lack of a decrease in escape‐flight speed during the 28‐day period, despite a gain in body mass, suggests a compensatory change in flight behavior. Indeed, all birds, independent of treatment, also slightly increased their wingbeat frequencies over time. This resulted in an average 1.2% increase in wingbeat frequency during the 28 days of experiments, which compensated any natural body mass gain‐induced increase in disk loading. As a whole, these results show that the increase in escape‐flight speed over time occurred via a behavioral adaptation in flight dynamics and not a change in body mass. This suggests that the birds improved their escape‐flight maneuverability via a skill‐learning process.

While birds had small adjustments on their behavior or body mass throughout the 28‐day period, tagged and untagged control birds did not significantly differ in how much they changed their wingbeat frequency or body mass (and consequent disk loading) over time. Therefore, over time there was no behavioral or body mass change that specifically compensated for the added effect of tag mass on escape‐flight speed.

In contrast, the untagged control birds increased their flight speed during the 28‐day experiment faster than the tagged birds, resulting in a 7% higher escape‐flight speed in the controls at the end of the 28‐day period. This suggests that tagged birds have a lower ability to increase their escape performance via skill learning, most likely due to detrimental tag effects. Alternatively, tagged birds might have limited their increase in escape performance to restrict the energetic costs associated with increasing flight speed (Norberg, 1981). Energy conservation might be particularly relevant in a natural setting when animals face harsh weather, unpredictable food availability and are exposed to predation risk (Broggi et al., 2021), yet the birds in our study had shelter and ad libitum food.

Our finding that there was no reduction in body mass to compensate for the added mass after tagging is in line with daily (Delingat et al., 2009; Kullberg, 1998; Meijer et al., 1994; Metcalfe & Ure, 1995) and seasonal (Macleod et al., 2008; Meijer et al., 1994) variation in body mass, reflecting their resilience to changes in added weight. Thus, also in natural contexts birds already are faced with the decision of the costs and benefits for survival and reproduction of carrying additional mass in terms of food, fat, or eggs throughout the day and seasons (Kullberg et al., 2002; Lind et al., 2010). This natural variation involves physiological changes and may take days to be completed (Eikenaar, 2017), for example when birds deposit fat before migration (Kullberg et al., 1996) or cold periods (Thomas, 2002). Tagging impacts may thus actually differ depending on the physiological state of the animal (Kullberg et al., 1996, 2002; Lind et al., 2010; Tomotani, Bil, et al., 2019), and this state may also affect whether or not animals can further adjust their gain/loss in mass after tagging.

In the present study, we did not observe a reduction in body mass to compensate for the added mass after tagging. This shows that one cannot assume that birds will always adapt their morphology or physiology to compensate for the mass increase due to tagging. Snijders, Nieuwe‐Weme, et al. (2017), using the same two types of tags as the ones in this study, showed that tags only impacted breeding when birds were captured and radio‐tagged in a sensitive period, that is, they found an effect during parental care, but not when they were radio‐tagged before starting to breed. Among birds which were tagged during parental care, parents with larger broods, and thus higher workload, and also females in lower body condition, were more likely to desert, indicating that effects of these radio‐tags on fitness depend on the moment of radio‐tagging and the already existing workload. Furthermore, in good years, parents tagged during the parental care period subsequently did not differ in nestling provisioning behavior compared with untagged birds. If these effects were primarily the result of the capture and tagging procedure or of the added tag weight remains unclear. In the future, it would also be interesting to determine whether such patterns are maintained in animals in different physiological states.

Finally, it is important to note that we used relatively heavy tags in comparison to those than are more and more often deployed in radio‐tagging studies. However, these tags have been successfully used in the wild and despite the use of those relatively heavier tags, the change in mass (and thus disk loading) caused by the radio‐tags was actually much less pronounced than the natural variation in mass within and between individuals.

### 4.2 Escape‐flight speed in tagged birds: not all effects are related to tag mass

Interestingly, the reduction in escape‐flight performance was not only caused by the added tag mass but we also revealed a tag mass‐independent effect. If tag mass would have been the only factor affecting escape speed, then the trend lines of flight speed versus disk loading would coincide for all treatments, which was not the case. Indeed, when tagged and untagged birds are compared, there was a significant difference in escape speed between tagged and untagged birds, independent on the mass of the tag. This suggests that there are factors other than the weight of the device that contributed to the decline in performance. A possible candidate is the harness that may limit leg push‐off dynamics during take‐off, a change in the center of gravity, or an overall “discomfort” of carrying a backpack.

These results are very similar to those described in a previous study that investigated the effect of carrying a tag, without an antenna, on the escape dynamics of blackcaps, performed in the same experimental setup as used here (Tomotani, Bil, et al., 2019). That study showed that the reduction in escape‐flight speed as a result of tagging can be modeled as Δ*U* = Δ*U*_tag_ + d*U*/d*m*∙Δ*m*, where Δ*U*_tag_ (=−0.08 m/s) is the mass‐independent effect of tagging on flight speed, and d*U*/d*m* (=−0.10 m/s/g) is the speed–mass slope, and Δ*m* tag‐induced mass increase (Tomotani, Bil, et al., 2019). The equivalently model for our study is Δ*U* = Δ*U*_tag_ + d*U*/d(*W*/*A*)∙Δ*W*_tag_/*A*, where Δ*U*_tag_ = −0.11 m/s and d*U*/d(*W*/*A*) = −0.11 m^2^ s/kg (Figure 4b). The mass‐independent effects on escape‐flight speed in our study on great tits were thus 34% larger than in the previous study on blackcaps. Based on d*U*/d(*W*/*A*) = −0.11 m^2^ s/kg for our study and the average actuator disk area in all here‐recorded flights (*A* = 110 cm^2^), the mass‐dependent effect of tagging on the escape‐flight speed of great tits is d*U*/d*m *= −0.12 m/s/g. Thus, for great tits the escape‐flight speed decreased 20% more per gram of tag mass than for the blackcaps. These combined results suggest that tagging affects the flight maneuverability in great tits more than in blackcaps, although differences in harness or tag type between the studies could also have affected this. Moreover, the radio‐tags in great tits had a standard radio‐tag antenna which might have affected flight behavior by touching the tunnel walls which may have led to additional adjustment sin flight behavior. The study on blackcaps used backpacks without such an antenna. A systematic study that directly compares species and harness and tag types and effects of antennas would be needed to determine this conclusively.

Despite the differences in magnitude, our model for predicting effects of carrying a radio‐tag on escape‐flight performance is applicable to great tits as well as blackcaps, two species with distinct ecologies. This suggests that the here‐described effect of tagging on escape‐flight maneuverability performance might be valid for a large range of passerine species. The similarity in model output also confirms the conclusion of Tomotani, Bil, et al. (2019), which was that if there is an interest of pre‐selecting of individuals to minimize the effect of tagging, one should tag the largest individuals, instead of the commonly used rule of tagging the heaviest individuals in order to minimize relative tag mass. This might be particularly important for birds that do not reduce mass in response to carrying a radio‐tag, as is the case in this study.

Our model based on actuator disk loading provides now also an estimate for determining what individual birds would be best for radio‐tagging studies. The individuals with the lowest disk loading have the highest escape‐flight speed, and thus for these individuals the tag‐induced reduction in flight speed might be least detrimental. The disk loading can be estimated based on body mass *m* and wing span *b* as *W*/*A* = *mg*/π*b* ^2^, and thus, when tagging birds one should select the largest and lightest fit individuals (the lowest body mass to wing span ratio). Both metrics that can be measured relatively easy in the field. We acknowledge, however, that our suggestions would only be feasible in studies where a large number of individuals are expected to be captured. Moreover, in several instances, the best design would be a randomized sampling of individuals, so results can be extrapolated to the population. However, if a selection has to be made, the largest (not heaviest) individual would probably also be the one least impacted by the tags.

Using the above‐defined model, we then simulated how the escape‐flight speed reduces after adding a fictitious tag of zero mass (Δ*U*_0%_ = −0.11 m/s), the here‐used lighter tag of ~5% body mass (Δ*U*_5%_ = −0.21 m/s), and the heavier tag of ~7% body mass (Δ*U*_7%_ = −0.25 m/s) (Figure 4b). This shows that for a bird with a disk loading of 18 N/m^2^ and a tag‐free escape‐flight speed of 3.10 m/s, the mass‐independent effect of tagging reduces escape‐flight speed with 4%, and the mass‐dependent effect of the lighter and heavier tag reduces this speed with an additional 3% and 5%, respectively. This highlights previous findings that mass‐independent effects such as attachment methods, general “discomfort,” and other factors are important when analyzing impacts of tags on birds (Blackburn et al., 2016; Bowlin et al., 2010; Chan et al., 2015; Snijders, Nieuwe‐Weme, et al., 2017; Tomotani, Bil, et al., 2019).

### 4.3 Integrating the effect of tagging on escape flight with radio‐tracking studies in the wild

We here used the escape‐flight performance as a proxy for rapid flight maneuverability, which can be relevant in many natural flight behaviors such as in‐flight hunting for flying insects, predator evasion, during social conflicts such as territory disputes or inter‐sexual interactions such as flight display. This is because acceleration and speed of all these types of flight maneuvers depend on the ratio between aerodynamic thrust force and body (+tag) weight, expressed by the actuator disk loading. Yet, given the large number of studies not finding reproduction or survival effects of radio‐tagging (Atema et al., 2016; Barron et al., 2010; Bell et al., 2017; Brlík et al., 2020; Snijders, Nieuwe‐Weme, et al., 2017), it would need to be determined in more detail if the here‐observed reduction in escape‐flight performance of tagged birds would indeed have an impact under natural condition.

Flying animals may rely on behavioral adjustments to cope with an increase in actuator disk load caused either by physiological changes or by device deployment. For example, the animal may stay more under cover and thus avoid having to make complex flight maneuvers during social conflicts or predatory interactions. Tagging may also impact the foraging ability of the bird, forcing individuals to adjust their foraging strategies or selection of food items. Given that few past studies found negative effects of radio‐tags on survival or reproduction, our findings suggest that tag effects could affect behavioral decision‐making in a more subtle way. Those subtle behavioral changes such as reducing risk‐taking behavior during foraging or social interactions can be difficult to quantify in the wild. Yet future studies could test this explicitly by comparing risk‐taking between tagged and untagged individuals, in playback or simulated predator exposure experiments.

Previous studies showed that songbirds with radio‐tags prospect novel environments widely in a biologically meaningful way (Amrhein et al., 2004; Roth et al., 2009), radio‐tagged resident birds maintain their home ranges and social interactions (Bircher et al., 2020, 2021; Naguib et al., 2001; Snijders et al., 2014), and migratory birds are capable of completing their migratory journeys (Bridge et al., 2013; Ouwehand et al., 2016; Peterson et al., 2015; Stutchbury et al., 2009; Tomotani, de la Hera, et al., 2019 and many others). Specifically, the studies using the same tags applied here provide a valuable comparison. Snijders et al. (2014) used the lighter tags from this study, revealing substantial movements across territories, showing personality‐dependent social network structures. Birds were tagged before breeding and the probability of breeding did not differ from untagged control birds, neither using the lighter tags (73% tagged birds found breeding at study site; 67% control birds found breeding; Snijders et al., 2014), nor using the heavier tags (74% tagged birds found breeding; Bircher et al., 2020). In a subsequent study using the heavier birds, tagging did not affect the likelihood of breeding (Snijders, van Oers, et al., 2017; Snijders, Nieuwe‐Weme, et al., 2017). Using the heavier tags, no indication was found that the tags resulted in low spatial activity. Both, Snijders, van Oers, et al. (2017) and Bircher et al. (2021) showed that tagged birds rapidly responded spatially to playback experiments and Bircher et al. (2020) quantified more than 30,000 forays by tagged birds into neighboring territories across the breeding season, reflecting a high movement across the season. Thus, any extra mass effects, while potentially present, do not appear to have compromised major spatial and social activities to be detrimental to behavioral studies. Because the tagged birds in our study did not compensate for the tag‐induced reduction in escape‐flight performance, this suggests that birds with radio‐tags compensate for it using a more complex behavioral response, such as by reducing risk‐taking or adjusting foraging strategies.

## 5 CONCLUSION

In conclusion, we show that a radio‐tag can significantly reduce the upward escape‐flight speed in an escaping passerine bird, and that during a period of one month, these birds did not adjust their flight behavior or body mass to compensate for the effect of the tag. In fact, the opposite dynamics was observed as untagged birds increased their upward escape‐flight speed over time faster than the tagged birds. This shows that adding a tag, such as the ones used here, to a songbird can affect its flight maneuverability at least for 28 days. Yet our results also confirm previous results showing that the tag mass alone is not a predictor of tag effects on escape‐flight speed (Tomotani, Bil, et al., 2019) or for behavioral effects in the wild (Snijders, Nieuwe‐Weme, et al., 2017). Given the common absence of a tag effect on reproduction and survival in songbird radio‐tagging studies, birds thus may adjust their risk‐taking behavior to avoid these rapid flight maneuvers. Our study thus supports previous suggestions that there are no simple effects of backpack tags on birds (Bowlin et al., 2010; Snijders, Nieuwe‐Weme, et al., 2017; Tomotani, Bil, et al., 2019). Effects depend on the mass and shape of the tag, the attachment method, the individual, the time of year, the ecological context, and the individual situation in which a bird is radio‐tagged. Moreover, we show here that tagging effects also vary with time after tag deployment, and that instead of observing a compensatory behavior in tagged birds, the detrimental effect of tagging is increased over time. These combined results show that only focusing on relative tag mass in guidelines for radio‐tagging studies is too simplistic and might even be erroneous (Tomotani, Bil, et al., 2019). A balanced assessment of the risks along with experience on a particular species seems more justified.

## CONFLICT OF INTEREST

The authors declare that there is no conflict of interest.

## AUTHOR CONTRIBUTION

**Barbara M. Tomotani:** data curation (equal); formal analysis (equal); investigation (equal); methodology (equal); software (equal); visualization (equal); writing—original draft (equal); writing—review and editing (equal). **Florian T. Muijres:** conceptualization (equal); data curation (equal); formal analysis (equal); methodology (equal); resources (equal); software (equal); writing—original draft (equal); writing—review and editing (equal). **Bronwyn Johnston:** investigation (equal); writing—review and editing (equal). **Henk P. van der Jeugd:** conceptualization (equal); funding acquisition (equal); resources (equal); writing—review and editing (equal). **Marc Naguib:** conceptualization (equal); methodology (equal); resources (equal); writing—original draft (equal); writing—review and editing (equal).

## Supporting information

MovieS1

MovieS2

## Data Availability

Data supporting this manuscript are available at the Dryad Digital Repository (https://doi.org/10.5061/dryad.12jm63xzw).

## 1

**TABLE A1:** Effects of treatment (control, heavier, or lighter tag), sex, and testing date on the bird disk loading calculated using bird mass or total mass (bird mass + tag mass). Statistics are given for the point of exclusion of the term from the model

	Estimate	*SE*	ndf	Ddf	*F*‐value	*p*‐Value
Disk loading (bird mass)						
Treatment: Sex: Date			2.00	43.26	1.14	.33
Treatment: Sex			2.00	11.99	6.29	.**01**
Treatment: Date			2.00	46.22	1.02	.37
Sex: Date			1.00	45.23	0.07	.79
Date			1.00	48.19	12.47	**<.01**
Treatment (Heavier): Sex (Male)	1.47	0.83				
Treatment (Lighter): Sex (Male)	−1.47	0.83				
Treatment (Control)	18.21	0.41				
Treatment (Lighter)	18.38	0.42				
Treatment (Heavier)	17.16	0.42				
Sex (Male)	−0.37	0.58				
Date	0.01	0.003				
Disk loading (total mass)						
Treatment: Sex: Date			2.00	43.24	1.14	.33
Treatment: Sex			2.00	12.00	6.25	.**01**
Treatment: Date			2.00	46.20	1.02	.37
Sex: Date			1.00	45.21	0.07	.79
Date			1.00	48.18	12.47	**<.01**
Treatment (Heavier): Sex (Male)	1.42	0.87				
Treatment (Lighter): Sex (Male)	−1.66	0.87				
Treatment (Control)	18.21	0.44				
Treatment (Lighter)	19.54	0.44				
Treatment (Heavier)	18.50	0.44				
Sex (Male)	−0.37	0.61				
Date	0.01	0.003				

Bold: *p*‐value < .05.

**TABLE A2:** Post hoc tests testing for the effects of treatment (with or without tag) or pairwise treatment comparisons on the effect of disk loading or flight speed. Statistics are given for the point of exclusion of the term from the model

	Estimate	*SE*	ndf	ddf	*F*‐value	*p*‐Value
Disk loading—bird mass (Tag–No Tag)						
Treatment: Date			1.00	47.08	0.001	.97
Treatment			1.00	15.95	2.28	.15
Sex			1.00	15.00	0.94	.35
Date			1.00	48.14	12.40	**<.01**
Date	0.01	0.003				
Flight speed (heavier–lighter)						
Treatment			1.00	9.96	0.87	.37
Flight speed (control–lighter)						
Treatment			1.00	10.00	1.51	.25
Flight speed (control x heavier)						
Treatment			1.00	9.74	15.78	**<.01**
Treatment (control)	3.09	0.04				
Treatment (heavier)	2.83	0.05				
Flight speed (Tag–No Tag)						
Treatment			1.00	15.71	5.04	.**04**
Treatment (No Tag)	3.09	0.07				
Treatment (Tag)	2.89	0.05				
Flight speed (Tag–No Tag)						
Treatment: Date			1.00	296.35	8.09	**<.01**
Disk loading (total mass)			1.00	25.23	18.95	**<.01**
Treatment (Tag): Date	−0.006	0.002				
Treatment (No Tag)	4.95	0.45				
Treatment (Tag)	4.93	0.47				
Disk Loading	−0.11	0.02				
Date	0.011	0.002				

Bold: *p*‐value < .05.

**TABLE A3:** Effects of treatment (control, heavier, or lighter tag), testing date or disk loading calculated using bird mass on flight speed. Statistics are given for the point of exclusion of the term from the model

Flight speed (bird mass)	Estimate	*SE*	ndf	ddf	*F*‐value	*p*‐Value
Treatment: Disk loading: Date			2.00	297.12	0.04	.96
Treatment: Disk loading			2.00	33.85	2.79	.08
Treatment: Date			2.00	297.61	5.49	**<.01**
Disk loading: Date			1.00	299.22	1.99	.16
Disk loading			1.00	27.97	14.74	**<.01**
Treatment (Heavier): Date	−0.004	0.003				
Treatment (Lighter): Date	−0.01	0.003				
Treatment (Control)	4.94	0.51				
Treatment (Lighter)	4.85	0.49				
Treatment (Heavier)	4.71	0.50				
Disk Loading	−0.11	0.03				
Date	0.01	0.002				

Bold: *p*‐value < .05.

**TABLE A4:** Effects of treatment (control, heavier, or lighter tag), testing date, or disk loading calculated using total mass (bird mass + tag mass) on flight speed. Statistics are given for the point of exclusion of the term from the model

Flight speed (total mass)	Estimate	*SE*	ndf	ddf	*F*‐value	*p*‐Value
Treatment: Disk loading: Date			2.00	297.61	0.03	.98
Treatment: Disk loading			2.00	34.01	2.58	.09
Treatment: Date			2.00	298.02	5.54	**<.01**
Disk loading: Date			1.00	299.25	1.94	.17
Disk loading			1.00	24.93	18.03	**<.01**
Treatment (Heavier): Date	−0.004	0.003				
Treatment (Lighter): Date	−0.01	0.003				
Treatment (Control)	4.98	0.47				
Treatment (Lighter)	5.01	0.48				
Treatment (Heavier)	4.90	0.49				
Disk Loading	−0.11	0.03				
Date	0.01	0.002				

Bold: *p*‐value < .05.

**TABLE A5:** Effects of treatment (control, heavier, or lighter tag), testing date, or disk loading calculated using bird mass or total mass (bird mass + tag mass) on wingbeat frequency. Statistics are given for the point of exclusion of the term from the model

	Estimate	*SE*	ndf	ddf	*F*‐value	*p*‐Value
Wingbeat frequency (bird mass)						
Treatment: Disk loading: Date			2.00	293.62	1.12	.33
Treatment: Disk loading			2.00	54.45	0.03	.97
Treatment: Date			2.00	295.86	1.81	.17
Disk loading: Date			1.00	297.02	0.14	.71
Treatment			2.00	14.92	2.13	.15
Disk loading			1.00	44.12	0.00	.97
Date			1.00	297.31	10.61	**<.01**
Date	0.01	0.004				
Wingbeat frequency (total mass)						
Treatment: Disk loading: Date			2.00	294.32	1.15	.32
Treatment: Disk loading			2.00	55.06	0.03	.97
Treatment: Date			2.00	296.01	1.81	.17
Disk loading: Date			1.00	297.05	0.14	.71
Treatment			2.00	14.92	2.13	.15
Disk loading			1.00	40.18	0.01	.93
Date			1.00	297.31	10.61	**<.01**
Date	0.01	0.004				
